# Type II enteropathy-associated T cell lymphoma in the duodenum: A rare case report

**DOI:** 10.1097/MD.0000000000020050

**Published:** 2020-06-05

**Authors:** Zhicheng Liu, Liang He, Yan Jiao, Helei Wang, Jian Suo

**Affiliations:** a Department of Gastroenterological Surgery; b Department of Hepatobiliary and pancreatic surgery, The First Hospital of Jilin University, Changchun, Jilin, China.

**Keywords:** lymphoma, enteropathy-associated T cell lymphoma, duodenum

## Abstract

**Introduction:**

Enteropathy-associated T-cell lymphoma (EATL) is a very rare form of lymphoma in the gastrointestinal tract. The proximal jejunum and ileum are the most common sites of EATL, whereas EATL rarely arises in the duodenum, and EATL involving metastasis of the bilateral ovaries is even rarer.

**Patient concerns:**

A 43-year-old female suffered from upper abdominal pain and weight loss for 3 months.

**Diagnosis:**

Type II EATL.

**Interventions:**

The patient was initially treated with chemotherapies, including 4 cycles of the CHOP-E and 2 cycles of the DHAP+ chidamide chemotherapy regimens. However, the patient did not respond well to chemotherapy. Surgical treatment of the duodenal obstruction, with perforation of small intestine and the duodenum, was performed successively.

**Outcomes:**

The patient died of septic shock only 1 day after the surgery for the second perforation. Her overall survival was 11 months from the time of initial diagnosis.

**Conclusion:**

This case suggests that EALT is highly invasive and its clinical course is very aggressive. Intestinal perforation, intestinal obstruction, or involvement of extraintestinal organs may occur in EALT patients. Additionally, EALT patients respond poorly to chemotherapy and have an extremely unfavorable prognosis.

## 1 Introduction

Enteropathy-associated T cell lymphoma (EATL) is a rare invasive lymphoma that originates from intraepithelial T lymphocytes of the gastrointestinal tract and is characterized by high invasiveness and an aggressive clinical course.^[1]^ According to the 2008 World Health Organization (WHO) classification and criteria for diagnosis, EATL is categorized into two major types: classical (type I EATL) and simplex (type II EATL).^[2]^ EATL can occur in any part of the gastrointestinal tract. The proximal jejunum and ileum are the most common sites of EATL, whereas EATL rarely arises in the duodenum or elsewhere in the small intestine.^[3]^ In addition, EATL with bilateral involvement of the ovaries is even rarer.^[3]^ Here, we report a case of a 43-year-old female patient with type II EATL arising primarily in the duodenum with bilateral involvement of the ovaries. The specific characteristics of this case may support a better understanding of this rare disease and improve our care for patients with similar clinical conditions.

## 2 Case presentation

A 43-year-old female suffered from upper abdominal pain and weight loss for 3 months. Over the previous 3 months, the patient experienced repeated abdominal pain and weight loss, accompanied by fatigue, without hematemesis, vomiting or fever. The patient had no significant past medical history or family history of malignancies.

Physical examination at the time of admission detected a mass above the navel approximately 9 × 6 cm in size with mild tenderness and nearly normal movement.

**Figure 1:**
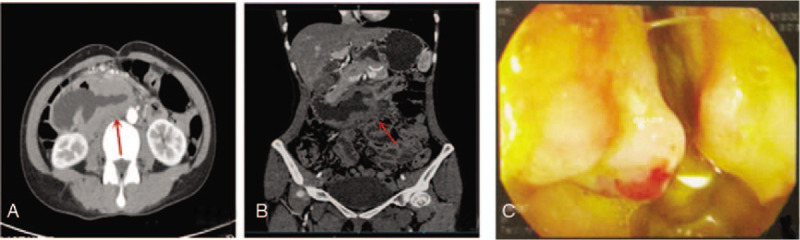
Imaging examinations. Abdominal CT images of transverse section (A) and coronal section (B) revealed space-occupying lesions in the pars horizontalis duodeni (red arrows), bowel-wall thickening, inhomogeneous enhancement, luminal dilatation. C, Gastroduodenoscopy showed irregular protuberant masses in the pars horizontalis duodeni.

**Figure 2:**
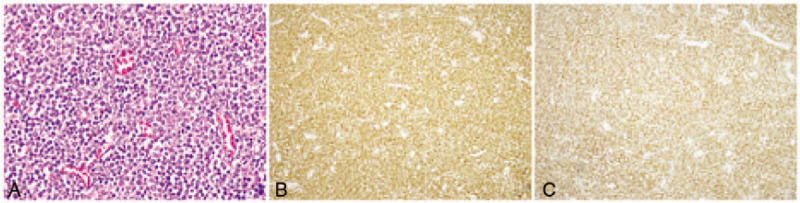
Immunochemical and immunohistological examinations of the tumor cells. A, At high magnification (H&E, ×400), the tumor cells were small with a similar shape and large, and deeply-stained nucleus; B, CD3^+^; and C, CD56^+^.

Biochemical examination found an erythrocyte sedimentation rate of 42 mm/h, lactate dehydrogenase activity of 131 U/L, and β2-microglobulin concentration of 1.93 mg/L. Abdominal enhanced computed tomography (CT) revealed an irregular wall thickening of the horizontal part of duodenum with uneven intensity and luminal dilatation (Fig. 1). Positron emission tomography (PET)-CT, an imaging technique with high value for the detection and staging of lymphoma, displayed thickening of the intestinal wall at the horizontal part of duodenum, and the radioactivity uptake was increased in an area of approximately 7.9 cm × 3 cm × 6.6 cm with a standardized uptake value as high as 7.9. The PET-CT findings indicated lymphoma in the patient. Gastroduodenoscopic examination showed irregular masses at the horizontal part of duodenum (Fig. 1C). Pathological examinations of bone marrow smears were performed and revealed excessive proliferation of marrow granulocytes, but a reduction in the production of erythroid cells. Immunohistochemistry analysis (Fig. 2). showed that the tissue biopsy was positive for CD4, CD8, CD43, CD56, granzyme B, and Ki67 (>50%), but negative for CD79a and TdT. In situ hybridization for Epstein–Barr virus-encoded small RNAs was negative, indicating no EB virus infection. Clonal rearrangement of T-cell genes showed T-cell receptor (TCR)β and TCRγ. Based upon the immunohistochemistry analysis, the patient was diagnosed as type II EATL.

The treatment process can be divided into the following 4 stages.

**Figure 3:**
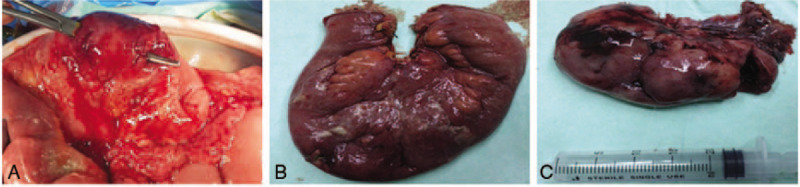
The enteric perforation and excised lesions in the patient. A, The 2 enteric perforations were found approximately 100 cm proximal to the ileocecal valve, and the lesions in the intestinal loop showed diffuse enlargement with a brittle texture. B, Excised fragment of the small intestine; and C, Excised left appendage.

1. The patient was initially treated with chemotherapies according to her will, including 4 cycles of the CHOP-E and 2 cycles of the DHAP+ chidamide chemotherapy regimens, and PET-CT was conducted to evaluate the clinical effectiveness of the chemotherapies. However, the results showed that chemotherapy was not effective and the tumor progressed (progressive disease, the max diameter increase more than 20% or new lesions develops).^[4]^
2. Laparoscopic gastrojejunostomy was performed because of duodenal obstruction 2 weeks after the end of chemotherapy. The patient refused further chemotherapy after the operation.
3. Partial enterectomy combined with enterostomy was performed because of intestinal perforation which may be caused by chemotherapy 5 months later. In addition, 2 cystic solid masses approximately the approximate 6 × 5 cm could be seen in the bilateral ovaries during the operation (Fig. 3). During the process of separation of adhesions in the surgery, the mass on the left ovary ruptured, making hemostasis difficult to achieve. For this purpose, left adnexectomy were performed. The patient was allowed to resume a liquid diet on the 6th day after the operation. Postoperative pathological examination revealed non-Hodgkin lymphoma EATL in the small intestine with bilateral metastatic ovaries.
4. The patient received an emergency operation again because of duodenal perforation on the 11th day after the first perforation. A perforation of the pars horizontalis duodeni was identified during the abdominal procedures, and repair of the perforation and abdominal drainage were performed.

Unfortunately, the patient died of septic shock only 1 day after the surgery for the second perforation. Her overall survival was 11 months from the time of initial diagnosis.

## 3 Discussion

EATL represents a rare form of non-Hodgkin lymphoma that accounts for only approximately 1% of all reported cases.^[5]^ The tumor cells in EATL are morphologically diverse, are medium to large in size, have round or polygonal nuclei, and have prominent nucleoli.^[6]^ The immunophenotype includes the following biomarker expression pattern: CD43^+^, TIA-1^+^, CD3^+^, CD5^–^, CD7^+^, CD8^±^, CD4^–^, CD103^+^, and TCR^±^. Intestinal perforation is one of the most common symptoms in the EATL patients and among the leading causes of death in these patients. For type II EATL, the tumor cells are small- to medium-sized, with darkly stained and round monomorphic nuclei, and with nuclear debris as well as necrosis. In addition, the tumor cells in type II EATL possess a unique immunophenotype, including CD3^+^, CD8^+^, CD56^+^, and TCR^+^ positivity and Epstein–Barr virus-encoded small RNAs negativity detected by in situ hybridization.^[2]^ The present case was classified as type II EATL based upon the clinical features, morphology of the tumor cells, and immunophenotype, which met the diagnostic criteria for type II EATL according to the 2016 WHO guidelines.^[5]^ In this most recently up-dated version, type II EATL is defined as monomorphic epitheliophilic intestinal T-lymphoma.^[5]^ Notably, the unusual site at the duodenum represents a unique feature of the presented case of type II EATL.

The patient was initially treated with chemotherapies, as generally recommended for gastrointestinal lymphoma. In fact, with the optimization of lymphoma chemotherapy regimens and the advent of targeted drugs, chemotherapies have been effectively used to achieve good clinical outcomes in patients with gastrointestinal lymphoma. In addition, the patients cannot well tolerate chemotherapy after surgery. Furthermore, lymphoma is considered a systemic disease, and chemotherapy is usually the first option while surgical resection is generally not recommended. As exceptions, surgical treatment is performed under the following conditions: persistent acute abdominal pain, suspected intestinal lymphoma, and unable to obtain a pathological diagnosis from endoscopy. However, Nijeboer et al^[7]^ demonstrated that the risk of death from perforation or obstruction in patients with EALT can be reduced by surgery contrary to other gastrointestinal lymphomas. For patients diagnosed with EALT, surgery should be performed in a timely manner or as soon as possible. Initial cytoreductive surgery combined with systemic chemotherapy is considered the standard treatment.^[6]^ In our case, the lesions were located in the pars horizontalis duodeni and were not identified in the initial gastroscopy in the local hospital. The patient firstly avoided the surgery because his pathologic examination was successfully achieved by an experienced endoscopist in our hospital. Otherwise, pancreatoduodenectomy would be performed, and the procedure-related life-threatening complication would occur in our patient.

Notably, the exact effectiveness of chemotherapy regimens in the treatment of EATL requires further prospective studies, mainly due to the low incidence of EATL. Therefore, standard chemotherapy regimens are still undetermined. Currently, the CHOP regimen is the most commonly used chemotherapy regimen for the treatment of EATL,^[6]^ with a total remission rate of as high as 46%,^[8]^ despite several disadvantages, including low tolerance and disease progression or digestive tract perforation during chemotherapy in a large proportion of patients. According to a number of previous studies, the prognosis of EATL is poor due to its low chemosensitivity, rapid growth, and tendency to transmit tumor cells, but can be improved by autologous stem cell transplantation.^[7,9]^ One study reported that the median overall survival and progression-free survival were 7 months and 1 month, respectively, with a 1-year survival rate as low as 36%.^[8]^

It was of note that the patient's disease in our study was insensitive to the CHOP and DHAP regimens, and the disease progressed during chemotherapy. Consequently, duodenal obstruction occurred, for which gastrointestinal anastomosis was performed. During the 5-month follow-up period, the patient underwent surgery for perforation of the small intestine due to the involvement of the lesion in the small intestine. Bilateral ovarian involvement was also found during surgery, which is one of the characteristics of this case, suggesting that EATL may metastasize to extraintestinal organs as well. Chan and colleagues indicated that the digestive tract and external organs, such as the omentum, pleura, pleural lymph nodes, supraclavicular lymph nodes, central nervous system, and breast, may be involved in type II EATL.^[10]^ Our case with bilateral involvement of the ovaries has not been reported previously, representing a new metastatic extra-intestinal organ rarely involved in type II EATL, may be a novel prognostic factor.

## 4 Conclusion

In summary, type II EALT in the duodenum is extremely unusual, with disease involving metastasis of the bilateral ovaries even rarer. Notably, EALT is highly invasive and its clinical course is very aggressive. Intestinal perforation, intestinal obstruction, or involvement of extraintestinal organs may occur in EALT patients. Additionally, EALT patients poorly respond to chemotherapy, and have extremely unsatisfactory prognosis.

## Acknowledgments

We thank the patient's husband for allowing us to using the data.

## Author contributions

Jian S was responsible for this study. Liu ZC, He L, and Wang HL were responsible for manuscript writing and literature review. Jiao Y was responsible for manuscript revising. All authors read and approved the final manuscript.

**Writing – original draft:** Zhicheng Liu, Liang He, Helei Wang.

**Writing – review & editing:** Yan Jiao, Jian Suo, Zou CC.
